# Encapsulated Pine Bark Polyphenolic Extract during Gastrointestinal Digestion: Bioaccessibility, Bioactivity and Oxidative Stress Prevention

**DOI:** 10.3390/foods10020328

**Published:** 2021-02-04

**Authors:** Pedro Ferreira-Santos, Raquel Ibarz, Jean-Michel Fernandes, Ana Cristina Pinheiro, Cláudia Botelho, Cristina M. R. Rocha, José António Teixeira, Olga Martín-Belloso

**Affiliations:** 1 Centre of Biological Engineering, Campus de Gualtar, University of Minho, 4710-057 Braga, Portugal; pedrosantos@ceb.uminho.pt (P.F.-S.); jmichel@ceb.uminho.pt (J.-M.F.); anapinheiro@deb.uminho.pt (A.C.P.); claudiabotelho@deb.uminho.pt (C.B.); cmrocha@ceb.uminho.pt (C.M.R.R.); 2 Agrotecnio Center, Department of Food Technology, University of Lleida, 25003 Lleida, Spain; raquel.ibarz@udl.cat (R.I.); olga.martin@udl.cat (O.M.-B.)

**Keywords:** *Pinus pinaster*, polyphenols, maltodextrin encapsulation, spray-drying, antioxidant activity, antibacterial activity, gastrointestinal digestion, oxidative stress

## Abstract

Polyphenolic extracts from pine bark have reported different biological actions and promising beneficial effects on human health. However, its susceptibility to environmental stresses (temperature, storage, etc.) and physiological human conditions prequires the development of efficient protection mechanisms to allow effective delivering of functionality. The aim of this work was to encapsulate pine bark extract rich phenolic compounds by spray-drying using maltodextrin, and understand the influence of encapsulation on the antioxidant and antimicrobial activity and bioaccessibility of phenolic compounds during gastrointestinal digestion. The optimized process conditions allowed good encapsulation efficiency of antioxidant phenolic compounds. The microencapsulation was effective in protecting those compounds during gastrointestinal conditions, controlling their delivery and enhancing its health benefits, decreasing the production of reactive oxygen species implicated in the process of oxidative stress associated with some pathologies. Finally, this encapsulation system was able to protect these extracts against acidic matrices, making the system suitable for the nutritional enrichment of fermented foods or fruit-based beverages, providing them antimicrobial protection, because the encapsulated extract was effective against *Listeria innocua*. Overall, the designed system allowed protecting and appropriately delivering the active compounds, and may find potential application as a natural preservative and/or antioxidant in food formulations or as bioactive ingredient with controlled delivery in pharmaceuticals or nutraceuticals.

## 1. Introduction

In recent years, an ever-increasing demand for supplements based on natural products has been recorded in Europe. In particular, thanks to the scientific evidences, many consumers believe that the consumption of plant-based supplements rich in particular phytochemicals can be useful for the prevention of some pathologies. This idea followed not only in the functionalization of foods and drinks with plant-based extracts but also strongly effected the supplement market, which recorded an exponential growth in the production and sale of dietary supplements, especially those plant-based [1,2].

*Pinus pinaster* L. bark, residue from the lumber industry, is highly rich in phenolic compounds, mainly including phenolic acids (e.g., ferulic, cinnamic and ellagic acids), flavonoids (e.g., taxifolin) and flavonols (e.g., narginin) [3]. It has been reported that pine bark extracts (PBE) have beneficial biological effects, including anti-inflammatory, antiviral, antitumor, antibacterial, antioxidant and therefore can be used as nutraceutical preparation or for the formulation of supplements [4,5]. In this sense, the PBE may become highly attractive for the food and pharmaceutical industries, as a potential functional ingredient. Several studies, demonstrated that *P. pinaster* bark (Pycnogenol^®^), reduces hyperpigmentation and improves the skin barrier function and extracellular matrix homeostasis [6], and shown beneficial effects in the prevention of several diseases, such as asthma, lupus erythematosus and cardiovascular diseases [1,7].

Phenolic compounds are very important molecules that not only act as antioxidants (donors of electrons that neutralize reactive oxygen species (ROS) and other free radicals) but also display several functions related to cell differentiation, deactivation of pro-carcinogens, maintenance and reparation of DNA, and other important actions [8,9]. Among the phenolic compounds, flavonoids, phenolic acids, stilbenes and tannins, especially proanthocyanidins, are particularly important [4,10]. Depending on their structure, phenolics may inhibit the growth and proliferation of certain cancer cells, and the effects are thought to be either direct, due to their electron and proton donor capacity, or indirect due to their ability to alter the activities of key enzymes in cellular response [10].

Furthermore, it is important to take in consideration that, the bioactive compounds of the PBE may suffer significant changes when exposed to adverse environmental (light, oxygen, temperature) and gastrointestinal (GI) conditions [11].

The in vitro GI models have been used to simulate the physiological conditions of the GI human tract and using a constant proportion of enzymes and salt concentrations, pH and digestion time for each digestive phase to faithfully re-create real-life conditions [12]. Static in vitro digestion models are now very well described and broadly used, once they present numerous advantages over in vivo and dynamic GI models [12]. Moreover, it allows to reach important conclusions about the bioavailability and bioaccessibility of several matrices, such as food, bioactive compounds, supplements and isolated molecules [13,14].

In this context, the phenolic compounds protection is of upmost importance. So, encapsulation has been the preferred method to protect bioactive compounds from oxidative processes. Additionally, the encapsulation of these moieties allows a controlled released of the molecules from the capsules during the digestion [15].

Spray-drying is the most common and cheapest technique to produce microcapsules when compared to other encapsulation methods [16]. To obtain high encapsulation efficiency and microcapsule mechanical stability, an optimization step is required taking into account the physicochemical properties of the core and wall materials, the spray-drying operation conditions (feed, air inlet and air outlet temperatures) and the desired functional properties of the final microcapsules [15]. Wall materials are particularly important in spray-drying. They should have sufficient solubility in the feeding liquid, film forming and emulsifying ability, as well as low viscosity at high concentrations. Maltodextrins (MD) are one of the main hydrolyzable carbohydrate-based wall materials used as encapsulating agents, having low cost and easily availability [17]. MD forms a coating film, which minimizes oxygen contact with the encapsulated materials and preserves them from other external agents, allowing a release under controlled conditions [18].

There are numerous research works on encapsulation of phenolic compounds derived from plant extracts by different methods (spray-drying, electrospinning, etc.) using several coating agents [11,19,20,21]; however no study was found on the encapsulation of phenolic PBE, promoting the protection and stability of bioactive compounds, and on the evaluation of their bioactivity and bioaccessibility.

The objective of the current study was to develop an effective protective system for pine bark extract, rich in phenolic compounds, by optimizing the spray-drying encapsulation process using MD as coating material. Additionally, it was proposed to determine the encapsulation influence on the bioaccessibility and bioactivity in terms of antibacterial and antioxidant activity of the PBE phenolic compounds. Its effect on colorectal adenocarcinoma cell line (Caco-2) viability and reactive oxygen species (ROS) production prior and after gastrointestinal digestion (GID) was also evaluated. The extracts and target bioactivities were chosen considering previous results from our research group [22], as PBE demonstrated high antioxidant, antidiabetic and antimicrobial activities, with low cytotoxicity, and potential to be used in food formulation and processing, either with a technological function (such as preservative or antioxidant) or as a bioactive ingredient in therapeutic formulations.

## 2. Materials and Methods

### 2.1. Raw Material and Chemicals

*Pine pinaster* bark (approximate age 15 years) was collected in Ponte de Lima, Portugal, in April 2016. The bark was washed, dried and milled to a granulometry of 1–1.6 mm. Maltodextrin with 14–17 dextrose equivalent Folin-Ciocalteu reagent, 2,2′-Azino-bis(3-ethylbenzothiazoline-6-sulfonic acid) diammonium salt (ABTS), 2,2-Di(4-tert-octylphenyl)-1-picrylhydrazyl (DPPH), 2,4,6-Tris(2-pyridyl)-s-triazine (TPTZ), 6-hydroxy-2,5,7,8-tetramethylchroman-2-carboxylic acid (Trolox), a-amylase (A1031, CAS 9000-90-2), pepsin (P7012; CAS 9001-75-6), pancreatin (P7545; CAS 8049-47-6), bile salts (B8631; CAS 8008-63-7), Dulbecco’s Modified Eagle Medium (DMEM), fetal bovine serum (FBS), penicillin-streptomycin solution, resazurin sodium salt, dimethyl sulfoxide (DMSO, ≥99.9%) and all standard markers for HPLC were procured from Sigma Aldrich (St. Louis, MO, USA). DCFDA/H2DCFDA-Cellular ROS Assay Kit (ab113851) was procured from Abcam plc (Cambridge, UK). All other chemicals used were of analytical grade and water was bidistilled.

### 2.2. Pine Bark Extract Preparation

The extraction methodology was previously optimized to maximize the extraction of phenolic compounds [22]. Briefly, 10 g of pine bark were mixed with 100 mL of water/ethanol (30:70, *v*/*v*), using cylindrical reactors protected from light, thermostatized at 83 °C, for 30 min under shaking (170 rpm). The mixture was centrifuged at 3000 rpm for 5 min and vacuum filtered. The extracts produced in the different batches, were combined and the solvent was evaporated at 40 °C using a rotary evaporator (Heidolph VV2000, Schwabach, Germany). The aqueous phase was lyophilized and extracts were stored at 4 °C until use.

### 2.3. Encapsulation Process by Spray-Drying

#### 2.3.1. Experimental Design

The lyophilized pine bark extracts (LPBE) was encapsulated with MD as wall material (shell). To select the optimal experimental conditions for the encapsulation process, a central composite design (CCD) according to a response surface methodology (RSM) was used. The independent variables were air inlet temperature (*T*, 140–180 °C), ratio of LPBE:MD (*r*, 1:25–1:35 *w*/*w*) and flow rate (*F*, 1–4 mL/min). The effect of the spray-drying conditions onto moisture content (MC, %) and encapsulation efficiency (EE) for the total phenolic content (TPC, %), and for the antioxidant activity (AA, %) by ABTS and FRAP assays, were evaluated. For the process, 1 g of LPBE was mixed with MD following the experimental design conditions shown in Table 1. The mixtures were homogenized at 1600 rpm for 5 min with an Ultra-Turrax T25-Basix mixer (IKA, Staufen, Germany). Spray drying was carried out in a Mini Spray Dryer Büchi model B-191 (Büchi Laboratoriums Technik, Flawil, Switzerland) using compressed air at 6 bar. All samples were atomized at a constant nozzle rate of 600 L/h and at maximum aspiration (100%).

A second-order polynomial model was proposed for each response, *Y_i_* (Equation (1)):

(1) $$Y_{i}=\beta_{0}+\sum_{i=1}^{k}\beta_{i}\;X_{i}+\sum_{i=1}^{k}\beta_{ij}X_{ij}^{2}+\sum_{i=1}^{k-1}\sum_{j>1}^{k}\beta_{ii}X_{i}X_{j}$$

where *Y_i_* were the dependent variables; *X_i_* and *X_j_* were the independent variables, *β*_0_ was a constant, *β_i_*, *β_ij_*, and *β_ii_* were regression coefficients; *k* is the number of the independent parameters.

*Design Expert DX 7.01* program (Stat Ease Inc., Minneapolis, MN, USA) was employed for the experimental design, data analysis and model building. The data were analysed using one-way analysis of variance (ANOVA) followed by t-Student test when a significant difference (*p* < 0.05) was found among the encapsulated sample means. Data were reported as mean ± standard deviation (SD).

#### 2.3.2. Optimization by RSM

According to the desirability approach method described by Derringer & Suich [23], the optimal encapsulation conditions were determined using as response the MC, and the EE’s and AA measured by ABTS and FRAP assays, as well as the model parameters determined in Experimental design section. *T*, *r* and *F* were studied at three different levels (−1, 0, 1). The highest desirability is determined assigning the highest level to the EE and the lowest level to the MC concentration and choosing as factor settings the studied parameters.

### 2.4. Moisture Content

The MC (%) was determined by gravimetry according to the *AOAC* (2002) official methods of analysis [24]. An amount of approximately 2.5 g of powder (LPBE-MD) was accurately weighed and dried in hot air oven at 105 ± 2 °C until a constant weight. The moisture content (%) was calculated as difference between initial weight and dried weight divided by the initial weight of the powder.

### 2.5. Soluble Solids Content

The soluble solids content was determined by refractometry (UNE-EN 12143) using a Atago RX-1000 digital-refractometer (Atago Co., Ltd., Tokyo, Japan) covering the measuring prism with the sample at room temperature (20 ± 0.2 °C). The results were expressed in °Brix. Bidistilled water, at the same room temperature, was used to calibrate the instrument.

### 2.6. Encapsulation Efficiency for TPC and AA

The EE was calculated according to Equation (2).

(2) $$\mathrm{EE}\;\left(\%\right)=\frac{\mathrm{encapsulated}\;\left(\mathrm{LPBE}-\mathrm{MD}\right)}{\mathrm{non}-\mathrm{encapsulated}\;\left(\mathrm{LPBE}\right)}\times 100$$

### 2.7. Structural Characterization

LPBE and encapsulated LPBE with MD (LPBE-MD) were added to aluminium pin stubs on a Phenom Charge Reduction Holder (CRH) at 10 kV and a spot size of 3.3. The samples were coated with 20 Angstrom Au and characterized using a desktop Scanning Electron Microscope (SEM) (Phenom ProX, Eindhoven, The Netherlands). All results were acquired using the ProSuite software. The particle size was measured from different zones of microscopic sections using Image J software (US National Institutes of Health, http://rsb.info.nih.gov/ij/).

For optical microscopy, samples were suspended in glycerol, observed using a microscope BX51 with DP72 digital camera (Olympus, Tokyo, Japan) at a magnification of 100X.

### 2.8. Encapsulated Extract Analysis

To determine the TPC and the AA for the encapsulates (LPBE-MD powders) obtained by the spray-drying process, the LPBE-MD powders were rehydrated to drive the same content of the soluble solids measured before the encapsulation process according to Ballesteros et al. [20].

#### 2.8.1. Total Phenolic Content (TPC)

TPC of the extracts was determined following the method based on the chemical reduction of Folin–Ciocalteu reagent [3]. For all analyses, 5 μL of extract (water or ethanol 50% for control) was mixed with 15 μL Folin−Ciocalteu reagent, 60 μL of Na_2_CO_3_ (75 g/L). The prepared solution was kept at 15 °C for 5 min. Absorbance was measured at 700 nm by an UV/vis spectrophotometer (Synergy HT, BioTek Instruments, Inc., Winooski, VT, USA). Gallic acid (0–500 mg/L) was used for calibration (*R*^2^ = 0.996). The results were expressed as milligrams of gallic acid equivalents (GAE) per gram of dry weight (mg GAE/g dw).

#### 2.8.2. Antioxidant Activity (AA)

The AA was measured with different assays varying their mechanisms of the antioxidant action:

The radical cation decolorization (ABTS^·+^) and 2,2-diphenyl-1-picrylhydrazyl radical scavenging activity (DPPH) were measured spectrophotometrically according to the methods described by Ferreira-Santos et al. [3]. Trolox (0–0.55 mmol/L) was used for calibration (*R*^2^ = 0.995). The results were expressed as millimols of Trolox equivalent per 100 g of dry weight of extract (mmol Trolox/g dw). Ferric reducing antioxidant power (FRAP) assay was determined using ferric sulphate (II) heptahydrate (0–2 mmol/L) for calibration (*R*^2^ = 0.992) [25]. The results were expressed as millimols of ferrous equivalent per 100 g of dry weight (mmol Fe^2+^/100 g dw).

#### 2.8.3. Antimicrobial Activity

Antimicrobial activity was determined using non-pathogenic strains of *Escherichia coli* 1.107 (Gram-negative) and *Listeria innocua* 1.17 (Gram-positive), from the stock cultures at the Food and Technology Department, University of Lleida. *E. coli* or *L. innocua* were inoculated into 40 mL of tryptone soy broth (Biokar Diagnostics, Beauvais, France) and incubated at 37 °C, 120 rpm for 11 and 15 h, respectively to obtain colonies in the stationary growth phase (10^8^–10^9^ CFU/mL) [26]. To determine the reduction in viable cells over time, for each bacterial culture, in 4.5 mL of sterile Mili-Q water, a 0.5 mL bacterial-aliquot was mixed with LPBE or LPBE-MD achieving a final concentration of 0.00001 g/mL of LPBE. Serial decimal dilutions were carried out and counts of *E. coli* and *L. innocua* were performed by spreading the inoculated solution on McConkey and Palcam (Biokar Diagnostics) agar plates, respectively. Water was used as a control. Surviving microorganisms were counted after incubation of agar plates at 37 °C for 24 h.

### 2.9. In Vitro Gastrointestinal Digestion

The GID was performed using the INFOGEST digestion standardized procedure [12,27], simulating the digestion in mouth (oral phase), stomach (gastric phase), and small intestine (intestinal phase).

The oral phase consisted in the addition of simulated salivary fluid (SSF) with α-amylase (75 U/mL) to the LPBE (40 mg/mL) or LPBE-MD (ratio of LPBE or LPBE-MD to SSF of 50:50 (*w*/*v*)). The mixture (pH 7) was gently stirred for 2 min at 37 °C. The LPBE-MD mixture presented the same initial content of phenolic compounds as the LPBE mixture, which was determined taking into account the EE results.

For the gastric digestion, simulated gastric fluid (SGF) with porcine pepsin (2000 U/mL) (ratio of LPBE or LPBE-MD to SGF of 50:50 (*v*/*v*)) was added to the oral phase. The pH was adjusted to 3 with HCl (6 M), and the mixture was incubated at 37 °C in a shaking water bath for 120 min at 60 rpm.

For the intestinal digestion, simulated intestinal fluid (SIF) with pancreatin (based on trypsin activity of 100 U/mL) and bile solution (10 mmol/L) was added to the gastric phase (ratio of SIF to the gastric chyme of 50:50 (*v*/*v*)). The pH was adjusted to 7 with NaOH (1 M), and the mixture was incubated for 120 min at 37 °C with stirring at 60 rpm.

After each step of the digestive process, a volume of 1 mL of the mixture was taken, centrifuged (10 min at 2700 *g*) and filtered (0.45 µm). In all fractions, the TPC and AA were determined as described in previous sections (Section 2.8.1 and Section 2.8.2, respectively).

### 2.10. Bioaccessibility of Phenolic Compounds

The bioaccessibility (%) was determined evaluating the effect of each digestion phase on the phenolic content, and was calculated according to the Equation (3) [13].

(3) $$Bioaccessibility\;\left(\%\right)=\left(\frac{A}{B}\right)\times 100$$

where *A* is the phenolic content (mg GAE/g dw) quantified at each digestion step, and *B* is the Total phenolic content in the extract before GID.

### 2.11. Individual Phenolic Compounds Determination and Quantification by UHPLC

The LPBE and LPBE-MD individual phenolic compounds were analysed by UHPLC-DAD as described by Ferreira-Santos et al. [22] in a Shimadzu Nexera X2 UPLC chromatograph equipped with Diode Array Detector (DAD). Separation was performed on a C18 column (2.1 mm × 100 mm, 1.7 μm particle size; from Waters) at 40 °C, using HPLC-grade water/formic acid (0.1%) and acetonitrile as eluents (0.4 mL/min). Phenolic compounds were identified at different wavelengths by comparing their UV spectra and retention times with that of the corresponding standards. Limit of detection (LOD) and Limit of Quantification (LOQ) were calculated using calibration curves of pure phenolic compounds standard, ranging between 250–2.5 mg/L (250, 125, 100, 50, 25, 10, 5, 2.5 mg/L) (Supplementary Materials Table S1). LOD and LOQ were calculated as previously reported [28]. In all cases, the coefficient of linear correlation was *R*^2^ > 0.99. Quantification was carried out using calibration curves and identified at different wavelengths (209–370 nm).

### 2.12. Biological Assays

#### 2.12.1. Cell Viability

The human colorectal adenocarcinoma Caco-2 cell line was kindly provided by Dr. Andreia Gomes (Department of Biology, University of Minho). Cells were maintained in a humidified atmosphere of 5% CO_2_ at 37 °C. Cells were grown in Dulbecco’s Modified Eagles medium (DMEM) supplemented with 10% foetal bovine serum, 1% non-essential amino acids and 1% penicillin/streptomycin.

When the cell culture reached 70–80% of confluence (confirmed by microscopic observation), the cells were trypsinized (0.25% trypsin-1 mM EDTA) and seeded in a 96-well plate at a density of 2 × 10^4^ cells per mL. The cell line was incubated with different cell culture medium: (i) supplemented DMEM; (ii) supplemented DMEM with undigested and (iii) supplemented DMEM with digested extracts LPBE, LPBE-MD and encapsulated agent (MD) in concentrations ranging from 75 to 2000 µg/mL for a period of 24 h. After incubation, the metabolic activity of Caco-2 cells (cell viability) was evaluated by the resazurin (7-Hydroxy-3H-phenoxazin-3-one-10-oxide sodium salt) reduction assay [22]. Briefly, the supernatant was replaced by 200 µL culture media containing resazurin (0.5 mM in PBS). After 2 h of incubation at 37 °C, 150 µL of the supernatant were transferred to a new 96-well microplate and the resultant fluorescent product (resorufin) was detected at 560 nm (λex) and 590 nm (λem) using a microplate reader (Cytation 3, BioTek Instruments, Inc., Winooski, VT, USA).

The % cell viability was calculated correcting blank values (cell-free medium) and related to untreated controls (0.5% DMSO).

#### 2.12.2. Measurement of Intracellular ROS Levels

Caco-2 cells were grown in 96-well plates at a density of 2.5 × 10^4^ cells per mL and were incubated overnight at 37 °C in a humidified atmosphere with 5% CO_2_. For treatment, extracts were dissolved on cell culture medium at a concentration of 500 μg/mL. Cells were incubated with the extracts for 8h. Then, cells were incubated for 45 min with 25 µM 2′,7′-dichlorofluorescein diacetate (DCFDA) at 37 °C in a humidified atmosphere with 5% CO_2_ and protected from light. For tert-butyl hydrogen peroxide (tbHP) ROS induction, the cell culture was then replaced by 100 µM tbHP (dissolved in Phosphate Buffer Solution (PBS)) except negative control cells, which were incubated with PBS. After 1h incubation protected from light, the fluorescence intensity was measured using a microplate reader (Cytation 3, BioTek Instruments, Inc., Winooski, VT, USA). Excitation and emission wavelength settings were 485 and 535 nm, respectively. The intensity of fluorescence is considered as a reflection of the total intracellular ROS levels.

### 2.13. Statistical Analysis

All experiments were performed in triplicate and the results are presented as average ± SD. GraphPad Prism^®^ software (San Diego, CA, USA) was used for statistical analyses. The level of significance was determined by one-way ANOVA followed by Bonferroni’s test for multiple comparisons. Significance was accepted at *p* < 0.05.

## 3. Results and Discussion

### 3.1. Spray-Drying Process Evaluation

#### 3.1.1. Moisture Content and Encapsulation Efficiency

Table 1 summarizes the results obtained from the CCD experimental design to optimize the spray-drying encapsulation process. The ANOVA, the significance of *p*-value, the determination coefficient (*R*^2^) and the adjusted determination coefficient (*R*^2^-*adj*) are shown in Supplementary Materials Table S2. To evaluate the model adequacy, the *R*^2^ and *R*^2^-*adj* values were used.

The effect of the temperature (*T*), the core to wall material ratio (LPBE:MD) (*r*) and the flow rate (*F*) were determining factors for the stability of the powder and the viability of the encapsulated pine bark phenolic compounds. The encapsulated powder *MC* is one of the most important variables to maintain the encapsulated products quality and stability [29]. The lowest *MC* (2.15%) for the spray drying encapsulation conditions was reached at the highest air inlet temperature (180 °C), the lowest ratio LPBE:MD (1:15) and lowest flow rate (1 mL/min). By the contrary, the highest *MC* (4.89%) was obtained when LPBE was encapsulated at 140 °C, with a 1:35 LPBE:MD ratio and flow rate of 4 mL/min (Table 1). The interactions flow rate-LPBE:MD ratio and temperature-LPBE:MD ratio as well as the temperature quadratic term significantly influenced on the *MC*. Thus, for spray drying encapsulation process, a flow rate and ratio core:shell increases, promote the rise of the final *MC*, while a temperature increase, reduces the final *MC*, wich is the desired situation in this case. The ANOVA data (Supplementary Materials Table S2) shows that the response functions obtained from the second-order model fitted correctly the experimental data (*p* < 0.01), where the determination coefficient (*R*^2^) was 0.906, meaning that the model was suitable for predicting the response variables. It is pertinent to point out that adjusted *R*^2^ (0.811) was lower than *R*^2^, suggesting that the model effect was significantly improved by the predictor.

The statistical analysis indicated that the second-order models were adequate for describing with accuracy the encapsulation efficiencies in terms of *TPC* (0.856), *AA_ABTS_* (0.934), and *AA_FRAP_* (0.933) (Supplementary Materials Table S2). Moreover, the adjusted determination coefficients 0.812 (*TPC*), 0.901 (*AA_ABTS_*), and 0.910 (*AA_FRAP_*) were lower than the corresponding *R*^2^. The maximal *EE* for *TPC* (79%) was reached when spray drying conditions were 160 °C, 1:25 LPBE:MD ratio, and 1 mL/min. The minimal *TPC* (29% and 30%) were reached at 140 °C, 1:35 LPBE:MD ratio and 180 °C, 1:15 LPBE:MD ratio, respectively, at the same flow rate (1 mL/min). The *TPC* decreased when temperature increased and ratio decreased and vice versa. Also, the interaction temperature- LPBE:MD ratio significantly influenced on the *TPC*. That is, as the ratio decreased, the effect of the temperature became more pronounced on TPC encapsulation efficiency.

For the *AA* measured by *ABTS* and *FRAP* assays, the highest *EE* were 120% and 114%, respectively, obtained at 180 °C, 1:15 LPBE:MD ratio, and 4 mL/min for both assays. The *AA* values suggest that the low LPBE:MD ratio used, together with the high flow rate, were not enough to coat the core, being not allowed to create a shell able to protect the phenolic pine bark compounds, which were susceptible to polymerization at the air inlet temperature used in the drying chamber. At temperatures higher than 80 °C, the Maillard reaction formed novel polymerized products contributing to the *AA* effect leading to values above 100% [30]. The air inlet temperature and LPBE:MD ratio significantly influenced on the *EE* of the *AA* (*ABTS* and *FRAP*) during the encapsulation process. *AA* increased with temperature (from 140 to 180 °C) and LPBE:MD ratio (from 1:15 to 1:35) maintaining the flow rate at 1 mL/min. Furthermore, the interaction of temperature and flow rate had a significant effect on *EE*. For example, when LPBE:MD ratio was set at 1:25 at temperature of 160 °C and flow rate from 1 to 4 mL/min, the *EE* augmented from 91% to 103% for *AA_ABTS_*, and from 91% to 96% for *AA_FRAP_*. In addition, when LPBE:MD ratio was set at 1:25 at a constant flow rate of 2.5 mL/min and the temperature ranged from 140 °C to 180 °C, the *EE* rises from 101% to 105%, and from 74% to 112% for *AA_ABTS_* and *AA_FRAP_*, respectively.

#### 3.1.2. Optimal Encapsulation Conditions and Model Validation

In the range of the studied spray-drying encapsulation conditions, an optimization was performed in order to find the combination of the spray drying conditions that gives the lowest MC, and the highest EE in terms of *TPC* and *AA*.

The optimum processing condition was achieved at a drying chamber temperature of 158 °C, with a ratio 1:18 [LPBE (core):MD (shell)], and with a flow rate of 0.815 mL/min. At this conditions, the lowest moisture content was 2.37% and the highest values for the EE were 67%, 98%, and 99% for *TPC*, *AA_ABTS_* and *AA_FRAP_*, respectively. The desirability of the spray-drying encapsulation treatment conditions was 0.835, which was taken as an indicator of accuracy between the polynomial model predictions and the experimental data (Table 1 and Supplementary Materials Table S2). The correlation coefficients between the measured and predicted values were 0.906, 0.856, 0.934, and 0.933 for *MC*, *TPC*, *AA_ABTS_* and *AA_FRAP_* models, respectively, indicating that the second-order expressions obtained for each assay (Supplementary Materials Table S2) adequately fitted experimental results.

The LPBE-MD ratio obtained at the optimal encapsulation conditions by spray drying was selected to evaluate their antimicrobial properties, in vitro stability and bioaccessibility in comparison to LPBE.

### 3.2. Structural Characterization

**Figure 1:**
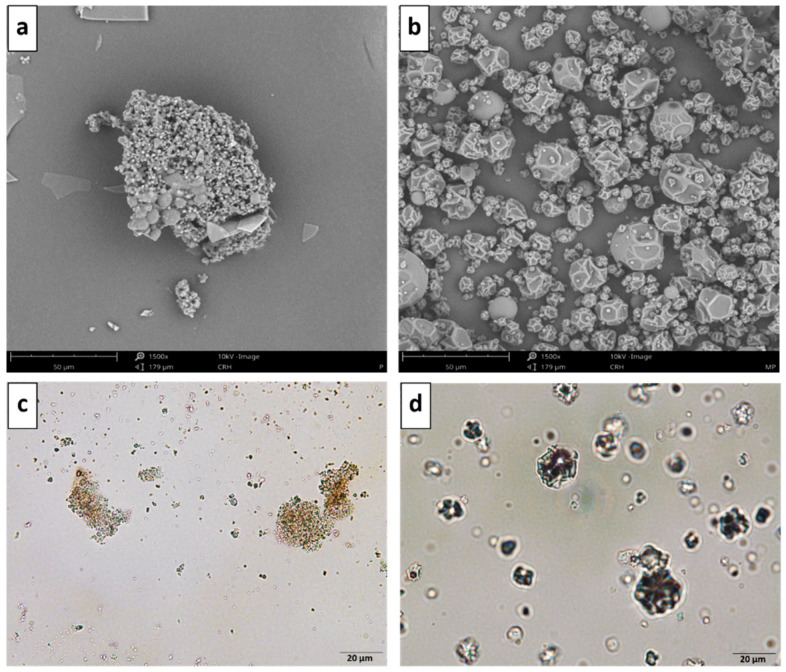
Images of SEM (**a**,**b**) and optical microscopy (**c**,**d**) of non-encapsulated LPBE (**a**,**c**) and encapsulated LPBE-MD (**b**,**d**) PBE. Scale bar of 50 µm and 20 µm applies to SEM and optical images, respectively.

In Figure 1, it can be observed the non-encapsulated LPBE (Figure 1a,c), and the MD encapsulated extract (Figure 1b,d) produced under optimized conditions (inlet temperature of 158 °C; ratio LPBE:MD of 1:18 and flow rate of 0.815 mL/min). The size of microcapsules was not uniform, varying from 5 to 25 µm. It can be preceived that spherical-shape microcapsules with rough surfaces were formed, indicating encapsulation and retention of this phenolic extract. This structure may be due to the water evaporation rates during the spray-drying process, using a temperature of 158 °C. Other works report similar structures and size of MD microcapsules, used to encapsulate other polyphenols extracts by spray-drying [31,32].

### 3.3. Antimicrobial Activity Evaluation

Table 2 shows a significant antibacterial activity of LPBE and LPBE-MD samples against the gram-positive bacteria (*L. innocua*), compared to gram-negative (*E. coli*). Our results are in agreement with Kotzekidou, Giannakidis, & Boulamatsis [33] and Oliveira et al. [34], who suggest that gram-positive microorganisms are usually more sensitive to antimicrobials such as polyphenols, than gram-negative microorganisms. In this sense, no inhibitory effect against *E. coli* was observed (Table 2) due to the resistance of gram-negative microbes to antimicrobials, attributed to the strong hydrophilicity of the outer membrane of the two-fold layer structure of the cell envelope of these bacteria, which became a strong barrier [35].

Moreover, it was observed that for the same concentration (0.00001 g/mL) of PBE, the inactivation of *L. innocua* was higher for the encapsulated antimicrobial agent (LPBE-MD) than for the non-encapsulated, since the microorganisms were already completely inactivated after 1 h. This behaviour could be attributed to the wall material used for the encapsulation. Thus, MD modifies the properties of the encapsulated material, affecting their size, geometry, surface morphology, favoring the solubilization of the lipophilic biocompounds (core), facilitating their diffusion through the single membrane of the gram-positive bacteria, enhancing the cellular uptake and promoting the *L. innocua* inactivation [34,36].

### 3.4. Effect of Simulated In Vitro Gid on Pine Bark Extracts

#### 3.4.1. Phenolic Compounds Determination and Bioaccessibility Analysis

**Figure 2:**
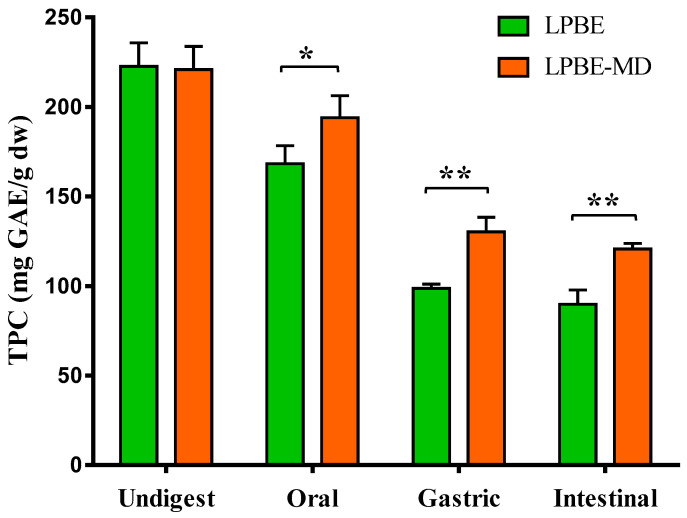
Total phenolic content (TPC, mg GAE/g dw) and bioaccessibility (%) of non-encapsulated (LPBE) and encapsulated (LPBE-MD) PBE before and after gastrointestinal digestion. Values are expressed as mean ± SD of three experiments. * *p* < 0.05, ** *p* < 0.01.

The impact of GID on TPC and bioaccessibility of LPBE and LPBE-MD is shown in Figure 2. The initial phenolic content is the same for LPBE and LPBE-MD with approx. 222 mg GAE/g dry extract. The results of the in vitro GID revealed that the TPC strongly decreased after the digestion in comparison to the undigested LPBE. When the extract was encapsulated (LPBE-MD), the degradation of these compounds was slower at the harsh conditions of the GI system (such as enzymes and pH) compared to the non-encapsulated extract (LPBE). 

In the oral phase, the content of phenolic compounds decreased, with a significantly higher reduction (*p* < 0.05) for the non-encapsulated extract when compared with the LPBE-MD (168.4 mg GAE/g and 193.9 mg GAE/g, respectively). In the gastric and intestinal phases, the TPC reduced approx. 20% more in LPBE compared to LPBE-MD. This study demonstrates that the phenolic compounds present in the extract have high instability when subjected to gastric conditions, which may be due to the presence of enzymes (such as pepsin) and the acidic pH of this phase. Other researchers also demonstrate that phenolic extracts are highly affected during the digestive process [14,37].

In this sense, the maltodextrin encapsulation of PBE significantly improved the bioaccessibility of phenolic compounds (Table 3). It can be observed that the encapsulation increased the bioaccessibility of phenolic compounds by approx. 11%, 15% and 14%, when subjected to the digestion conditions of the oral, gastric and intestinal phases, respectively.

The phenolic profile of LPBE and LPBE-MD before and during GID was tentatively identified and quantified by UHPLC-DAD (Table 4). Catechin, epicatechin/*p*-coumaric acid, gallocatechin, narginin, hesperidin and taxifolin were the most abundant phenolic compounds identified in the LPBE, showing values between 125–199 mg/L with the exception of taxifolin having a higher concentration (344 mg/L). Other flavonoids, hydroxybenzoic acids, hydroxycinnamic acids and stilbens (resveratrol) were identified with concentrations between 44 to 94 mg/L, except for apigenin and cinnamic acid, which had lower concentrations. Quercetin and gallic acid were identified in all samples, however, they co-eluted.

The individual phenolic concentrations of the PBE were significantly reduced after in vitro GID, in both LPBE and LPBE-MD. However, a more pronounced decrease in concentration was observed in the gastric and intestinal phase of GI digestion.

When the extracts were subjected to the GID, it was observed that, at the end, the composition of the encapsulated extract (LPBE-MD) was similar to the non-encapsulated extract (LPBE). Resveratrol, rosmarinic acid and gallocatechin are the less stable compounds, not being detected at the end of digestion. Other compounds such as taxifolin, 3,4-hydroxybenzoic acid, narginin, o-coumaric acid and cinnamic acid, are also partially destroyed or converted during the intestinal phase, having concentrations below of 53 mg/L, 12 mg/L, 5.2 mg/L, 4.9 mg/L and 2 mg/L, respectively. In addition, ferulic acid, chlorogenic acid and epicatechin/*p*-coumaric acid appear to be the most stable phenolic compounds to digestion, in accordance with results reported previously by Frontela et al. [38].

These results are important to unveil the bioavailability of the phenolic compounds present in the PBE, their biological activities and possible applications as nutrients and health promoters.

#### 3.4.2. Antioxidant Activity

**Figure 3:**
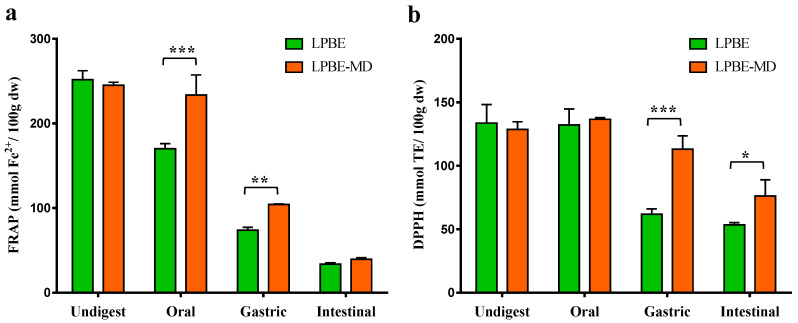
Antioxidant activity by FRAP (**a**) and DPPH (**b**) of non-encapsulated (LPBE) and encapsulated (LPBE-MD) PBE before and after gastrointestinal digestion. Values are expressed as mean ± SD of three experiments. * *p* < 0.05, ** *p* < 0.01, *** *p* < 0.001.

The influence of GID on the antioxidant activity of the encapsulated and non-encapsuled PBE is shown in the Figure 3. The reduction power (FRAP) of LPBE significantly decreased (*p* < 0.01) during the different stages of GID, and scavenging activity significantly decreased after the gastric and intestinal digestion phases (*p* < 0.01) in comparison to the undigested PBE.

When the AA was determined by the FRAP assay (Figure 3a), a significant decrease (*p* < 0.001) was observed in the bioactivity of the LPBE soon in the oral phase compared to the undigested extracts. This reduction was noticeably lower in the LPBE-MD, indicating a high protection level of the microcapsules for reduction AA in oral phase. Also in the gastric phase, the microcapsules showed a protective effect of the phenolic compounds, with significant differences between the LPBE and LPBE-MD extracts (*p* < 0.01). This protective effect was observed in a lesser extent in the intestinal phase.

In agreement with the TPC content, the DPPH results (Figure 3b) demonstrated that, after the digestive process, the encapsulated extract (LPBE-MD) shows a smaller reduction in its AA when compared to the non-encapsulated extract (LPBE). It can also be observed that, in the oral phase, the encapsulated and non-encapsulated extracts exhibit scavenging activity similar to the undigested extract. These results may lead to state that phenolic compounds that have been degraded do not have relevant AA. The differences are visible in the gastric and intestinal phase, where the LPBE-MD shows 46% and 30% more AA, respectively, compared to the LPBE. These results may be related to the marked decrease in the concentration of certain phenolic compounds with high AA during digestion, such as taxifolin, gallocatechin, resveratrol and rosmarinic acid.

Our data suggest that the AA of natural extracts is the result of the synergistic effects of the different phenolic compounds among each other and with other components of the matrix or the organism such as proteins, carbohydrates and lipids [39]. It has been reported that the AA of phenolic extracts from plant matrices are negatively affected when subjected to the conditions of the GID [14,39]. In a study, Frontela et al. [38] reported that the enrichment of fruit juices with pine bark phenolic extracts is an expedient strategy to compensate possible phenolic loss through gastrointestinal processing. The present study proves that these extracts can be even more efficient if incorporated in an encapsulated form.

#### 3.4.3. Cellular Viability and Antioxidant Capacity on Intestinal Caco-2 Cells

**Figure 4:**
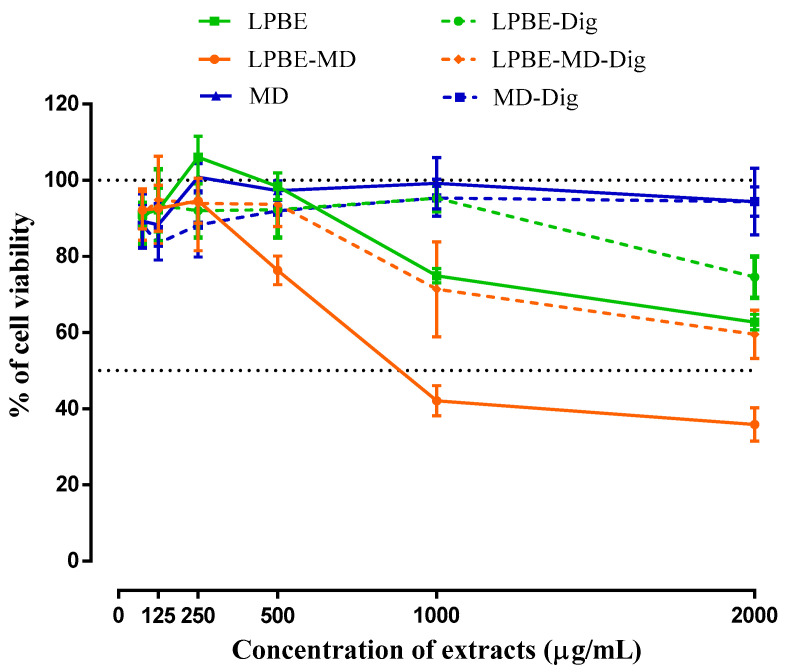
Cellular viability (%) of non-encapsulated (LPBE), encapsulated (LPBE-MD) PBE and maltodextrin (MD) as wall material before and after gastrointestinal digestion against human colorectal adenocarcinoma Caco-2 cells.

The Figure 4 represents the cellular viability in the presence of encapsulated (LPBE-MD) and non-encapsulated (LPBE) phenolic extract when subjected (or not) to the conditions of the GID system. Different concentrations of bioactive extracts (0 to 2000 µg/mL) were incubated with the Caco-2 cells for 24 h and their ability to metabolize resazurin into resorufin in the presence of extracts was used as a measurement of cells metabolic activity.

Our data shows that MD as wall material was not toxic toward Caco-2 cells, regardless of the concentration tested. On the other hand, the cells exposed with the LPBE non-encapsulated and/or encapsulated with MD, at concentrations higher than 500 µg/mL, show a significant reduction of cell viability in Caco-2 cells. In addition, when the PBE is encapsulated and the cells are exposed to the same concentration, the number of viable cells suffered a greater reduction of viability, compared to the non-encapsulated extract (LPBE).

Furthermore, Caco-2 cells were treated with a bioaccessible fraction of PBE resulting from the simulated GID. The results showed that cell viability decreased significantly when the cells were treated with both digested PBE (LPBE-Dig or LPBE-MD-Dig). This reduction was significantly greater for the encapsulated extracts, despite a smaller reduction compared to undigested extracts.

These results are in agreement with those discussed above, and confirm the protection of phenolic compounds by encapsulation and the promotion of a controlled release, protecting their biological activity and health benefits (e.g., anti-proliferative), as well as it can contribute to better sensory properties of encapsulated products.

As it is widely described, cancer development has many steps, from initiation and promotion to progression [40]. On this study it was evaluated the influence of bioactive compounds on the latest stage the progression. The literature describes several chemopreventive mechanisms induce by the presence of polyphenols, being one of them the ability to inhibit or reduced the proliferation of cancer cells [41,42].

When analyzing the composition in terms of phenolic content of the digested PBE it can observed that the compounds with higher bioavailability for the cells were catechin, taxifolin, ferulic acid and epicatechin/*p*-coumaric acid. Although, it was not possible to isolate the effect of each compound on the anti-proliferation ability of the PBE, it is believed that they may have a synergistic effect based on previous studies found in literature. It is know that the ferrulic acid inhibits osteosarcoma cells by interfering with the cell cycle as well as by inducing a significant increase on cell apoptosis [43]. Janicke et al. described that *p*-coumaric acid has a negative effect on colon cancer progression as it retards cell cycle progression in Caco-2 cells [44]. Similarly, taxifolin demonstrated that it can inhibit tumor growth in vivo, and it has been reported that this maybe due to cell cycle arrest in G1 [45].

Considering these results of low toxicity, LPBE concentrations of 500 µg/mL and their equivalent amount in LPBE-MD were chosen for the following antioxidant activity studies in Caco-2 cells.

Moderate levels of ROS are essential for cellular proliferation, differentiation, and survival. At the same time, and assuming the fundamental role of ROS at the beginning and its influence in a number of diseases, alternative forms have been studied to prevent their increase in the body [10]. To overcome cell death induced by oxidative stress, increasing antioxidant defenses has been an interesting therapeutic approach [46]. Extracts obtained from bioresources (plants, algae, agro-food wastes, etc) have the ability to change cell redox homeostasis, either by increasing or decreasing ROS production. Given previously obtained results of PBE composition (Figure 2 and Table 4) and promising results by FRAP and DPPH antioxidant assays (Figure 3), Caco-2 cells were selected to further elucidate the mechanism of extracts action in terms of antioxidant behavior. This cell line was used taking into account the results of cell viability and because they are widely used to understand the influence of nutrients and/or nutraceuticals in the body after ingestion.

**Figure 5:**
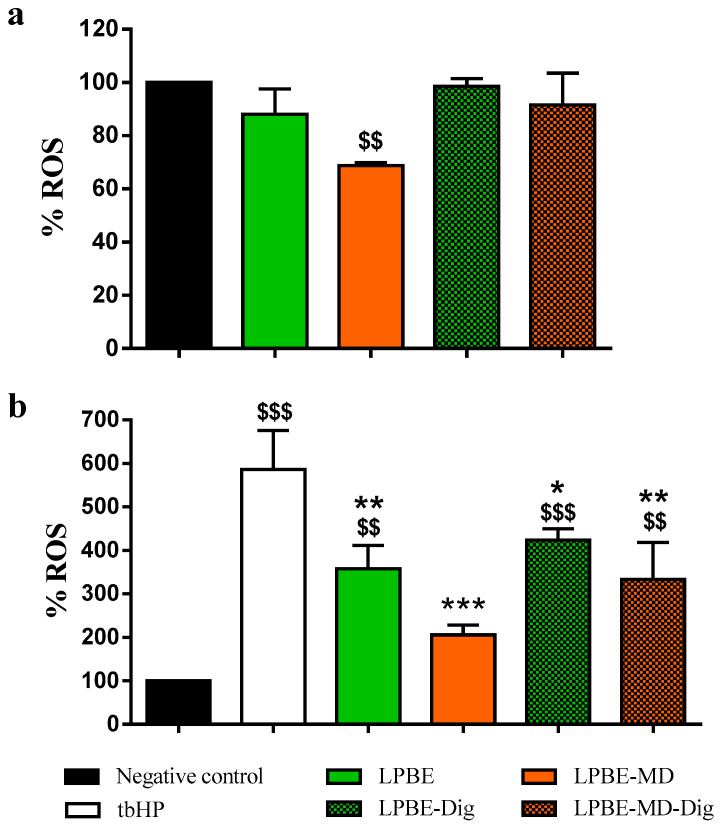
Antioxidant activity of extracts on Caco-2 cells. (**a**) Measurement of reactive oxygen species levels after 8 h incubation with 500 μg_extract_/mL of encapsulated/non-encapsulated (digested and undigested extracts). (**b**) Measurement of reactive oxygen species levels after 8 h incubation with 500 μg_extract_/mL of encapsulated/non-encapsulated (digested and undigested extracts) and further tert-butyl Hydrogen Peroxide insult (1h incubation with 100 mM tbHP). Values are expressed as mean ± SD of three experiments. ^$$^ *p* < 0.01, ^$$$^ *p* < 0.001 versus control cells, and * *p* < 0.05, ** *p* < 0.01, *** *p* < 0.001 versus tbHP-treated cells.

The level of ROS produced by Caco-2 cells after 8 h incubation with encapsulated and non-encapsulated LPBE before and after GID is shown in Figure 5. In the Figure 5a it is shown the influence of extracts on endogenous oxidative stress in Caco-2 cells. It is known that cancer cells have an altered redox state, expressing higher amounts of ROS that non-cancerous cells. The results showed that the PBE, when encapsulated, presented a significant reduction of endogenous intracellular ROS (about 34%) in the cancer cells used in this work. Figure 5b shows AA of the PBE when Caco-2 cells were subjected to exogenous oxidative stress caused by tbHP, which displayed a greater increase in ROS levels when compared to the control cells (4.8 times more). Moreover, when the cells were incubated with a concentration of 500 µg/mL of extract, the production of ROS induced by tbHP was significantly lower (approx. 30% lower than the control tbHP), confirming its antioxidant capacity. Our results are in agreement with those reported by Gascón et al. [10], wherein PBE from three different species (including *Pinus pinaster*) showed antioxidant activity in Caco-2 cells using concentrations above 20 µg/mL.

As previously evaluated by the in vitro antioxidant methods to evaluate iron reduction (FRAP) and scavenger activity (DPPH), it is clear that the digestion process influences the AA of PBE and the maltodextrin encapsulation process partially promotes the stabilization of bioactive phenolic compounds, preventing their degradation. This decrease in the antioxidant activity of the extract after digestion is considered normal, due to the degradation or (bio)tranformation of the phenolic compounds present in the bioactive extract. These data corroborate the results obtained by the intracellular ROS assay in Caco-2 cells in which the digested extracts had less preventive effect of ROS generation than the undigested extracts, despite the cells treated with the encapsulated extracts (LPBE-MD-Dig) show a lesser increase in intracellular ROS caused by tbHP.

## 4. Conclusions

The results obtained in this study demonstrated that pine bark extracts can be effectively encapsulated in maltodextrin through spray-drying, resulting in low moisture content and high encapsulation efficiencies. The structural analysis of the encapsulated extract highlighted the presence of spherical-shape microcapsules containing pine bark phenolic compounds. The microcapsules obtained under the optimal conditions showed a high efficiency in the encapsulation of bioactive compounds, preserving the high antioxidant and antimicrobial activity of these extracts. Moreover, the encapsulation demonstrated a protective effect on the phenolic compounds degradation (increased stability and bioaccessibility) during the digestion process, in particular in the oral and gastric phase of the gastrointestinal system. Finally, the maltodextrin encapsulation system was able to partially protect the extracts until the intestinal phase, potentiating effective delivery of the pine bark functionality in this final phase, where the phenolic compounds are absorbed. Pine bark extracts also provided efficient protection of Caco-2 cells against oxidative stress, and their antioxidative activity was positively affected by encapsulation, increasing the possible therapeutic effects. Furthermore, as the encapsulation process was particularly efficient in protecting the extracts in a highly acidic environment, this system will be suitable for the enrichment of fermented foods or fruit-based beverages, among other foods. Concomitantly, aiming at food safety, the encapsulated extract showed high efficiency in protection against *L. innocua*, making it useful in protecting foods susceptible to this type of contamination. Overall, this system allows increased feasibility of the pine bark extracts by protecting and appropriately delivering the active compounds, and may find potential application as a natural preservative and/or antioxidant in food formulations or as bioactive ingredient with controlled delivery in pharmaceuticals or nutraceuticals.

## Figures and Tables

**Table 1:** Experimental design for the independent variables and corresponding response values (measured and predicted) for spray drying encapsulates from pine bark by-product extracts.

	Independent Variables			Response Values							
				*MC* (%)		*EE* (%)					
						*AA_ABTS_*		*AA_FRAP_*		*TPC*	
Assay *	*T* (°C)	*r* (-)	*F* (mL/min)	Measured	Predicted	Measured	Predicted	Measured	Predicted	Measured	Predicted
**1**	160	1:25	4	3.01 ± 0.02	3.04	103.06 ± 11.62	87.08	96.49 ± 4.82	89.90	64.33 ± 6.21	72.78
**2**	160	1:25	2.5	2.91 ± 0.15	3.12	99.24 ± 54.36	92.78	94.58 ± 2.91	89.73	49.84 ± 12.34	62.38
**3**	160	1:25	1	2.73 ± 0.06	2.82	91.19 ± 5.84	99.48	91.38 ± 1.23	98.56	78.69 ± 9.84	64.48
**4**	160	1:25	2.5	3.19 ± 0.05	3.12	51.84 ± 28.71	92.78	96.59 ± 5.36	89.73	58.55 ± 3.29	62.38
**5**	140	1:35	4	4.89 ± 0.04	4.84	111.33 ± 9.27	99.39	68.15 ± 2.18	65.49	52.52 ± 1.41	51.54
**6**	160	1:25	2.5	3.47 ± 0.25	3.12	110.70 ± 12.58	92.78	87.60 ± 2.37	89.73	67.19 ± 3.27	62.38
**7**	180	1:15	1	2.15 ± 0.03	3.44	54.24 ± 36.84	106.49	73.98 ± 5.12	82.13	30.47 ± 1.19	30.02
**8**	140	1:15	1	3.77 ± 0.13	3.58	58.05 ± 11.43	72.84	55.71 ± 4.76	62.59	34.67 ± 1.36	36.46
**9**	180	1:25	2.5	3.96 ± 0.18	3.71	105.46 ± 7.05	95.54	112.03 ± 2.38	109.26	32.48 ± 7.59	43.68
**10**	160	1:25	2.5	3.35 ± 0.16	3.12	93.87 ± 1.23	92.78	90.63 ± 1.31	89.73	67.60 ± 3.74	62.38
**11**	180	1:35	2.5	2.69 ± 0.05	2.76	96.98 ± 10.91	110.69	112.75 ± 15.68	137.05	54.75 ± 1.25	53.41
**12**	180	1:35	4	3.99 ± 0.11	4.14	91.41 ± 11.34	76.23	126.58 ± 1.35	125.07	48.84 ± 1.98	46.80
**13**	140	1:15	4	2.74 ± 0.07	2.64	98.55 ± 10.04	83.97	94.06 ± 13.43	76.25	57.50 ± 1.35	59.24
**14**	180	1:15	4	3.13 ± 0.02	3.10	120.37 ± 22.58	86.68	113.57 ± 13.94	93.79	34.36 ± 4.16	28.84
**15**	140	1:25	2.5	3.77 ± 0.26	4.13	99.73 ± 7.85	90.02	74.44 ± 3.15	70.20	55.93 ± 4.87	49.16
**16**	160	1:25	2.5	2.94 ± 0.12	3.12	92.98 ± 2.28	92.85	81.79 ± 6.25	89.73	69.86 ± 5.25	62.38
**17**	160	1:15	1	2.29 ± 0.20	2.58	119.15 ± 23.06	88.46	84.65 ± 3.68	79.19	44.29 ± 2.84	48.10
**18**	140	1:35	1	4.07 ± 0.01	4.06	101.24 ± 2.04	103.71	52.30 ± 17.65	77.47	28.68 ± 3.18	38.98
**19**	160	1:35	2.5	3.51 ± 0.12	3.34	109.61 ± 7.63	97.79	96.64 ± 3.42	101.27	55.28 ± 1.27	56.06

* Assay order was randomized. *T*, temperature (°C); *r*, ratio lyophilized pine bark extract:Maltodextrin (-); *F*, flow rate (mL/min); *MC*, moisture content (%); *EE*, encapsulation efficiency (%); *TPC*, total phenolic content (%); *AA*, antioxidant activity measured by *ABTS* assay (%) and *FRAP* assay (%).

**Table 2:** Antimicrobial activity of non-encapsulated (LPBE) and encapsulated (LPBE-MD) PBE against *Escherichia coli* and *Listeria innocua*.

	LPBE (0.00001 g/mL PBE)		LPBE-MD (0.00001 g/mL PBE)	
Time	log *N*		log *N*	
	*E. coli*	*L. innocua*	*E. coli*	*L. innocua*
Control (0 h)	8.974 ± 0.584	7.867 ± 0.313	8.974 ± 0.584	7.927 ± 0.601
1 h	uncountable	2.662 ± 0.027	uncountable	<1
24 h	uncountable	<1	uncountable	<1

Data shown are a mean ± SD of three experiments. LPBE, lyophilized pine bark extrac; PBE, pine bark extract; MD, maltodextrin; *N*, number of microorganisms.

**Table 3:** Bioaccessibility (%) of non-encapsulated (LPBE) and encapsulated (LPBE-MD) PBE before and after gastrointestinal digestion.

	LPBE			LPBE-MD		
**Bioaccessibility** (%)	**Oral**	**Gastric**	**Intestinal**	**Oral**	**Gastric**	**Intestinal**
	75.7 ± 4	43.2 ± 2	40.4 ± 3	87.1 ± 5	58.5 ± 3	54.3 ± 1

Values are expressed as mean ± SD of three experiments.

**Table 4:** Phenolic compounds identification and quantification of non-encapsulated (LPBE) and encapsulated (LPBE-MD) PBE before and after gastrointestinal digestion by UHPLC-DAD.

	LPBE				LPBE-MD			
Compounds	Undigested	Oral	Gastric	Intestinal	Undigested	Oral	Gastric	Intestinal
catechin	198.2 ± 28.0	153.1 ± 14.9	142.3 ± 13.0	115.3 ± 0.8	162.2 ± 16.0	128.6 ± 11.2	135.5 ± 13.9	117.4 ± 0.4
vanilic acid	64.5 ± 6.5	55.8 ± 2.3	44.1 ± 2.8	44.3 ± 1.3	60.6 ± 8.1	59.2 ± 4.0	44.3 ± 2.1	44.1 ± 0.5
gallic acid	n.q.	n.q.	n.q.	n.q.	n.q.	n.q.	n.q.	n.q.
epicatechin + *p*-coumaric acid	127.7 ± 16.8	105.6 ± 16.7	89.2 ± 10.6	75.5 ± 3.1	131.2 ± 3.9	121.7 ± 8.3	94.0 ± 9.0	117.7 ± 6.4
*o*-coumaric acid	57.8 ± 7.8	61.2 ± 30.4	31.3 ± 0.3	7.4 ± 3.4	63.9 ± 8.9	65.7 ± 11.2	64.5 ± 28.6	4.9 ± 0.1
chlorogenic acid	44.1 ± 4.0	35.6 ± 1.1	30.9 ± 1.8	32.4 ± 0.6	41.4 ± 1.5	41.5 ± 1.7	33.4 ± 2.0	32.1 ± 0.4
ferulic acid	48.7 ± 9.9	29.6 ± 4.2	35.1 ± 3.5	31.9 ± 1.2	58.7 ± 9.9	60.2 ± 5.6	54.1 ± 4.7	51.9 ± 0.5
ellagic acid	62.1 ± 12.6	47.9 ± 12.6	34.4 ± 2.1	23.9 ± 3.4	62.1 ± 12.6	55.8 ± 6.4	57.6 ± 8.6	27.0 ± 5.8
narginin	164.0 ± 23.4	134.5 ± 31.0	97.2 ± 0.9	6.8 ± 2.7	170.2 ± 21.3	140.3 ± 33.1	117.1 ± 38.7	5.2 ± 0.1
hisperidin	125.9 ± 23.1	84.6 ± 18.9	67.1 ± 10.3	30.0 ± 2.1	135.0 ± 18.0	115.4 ± 9.8	86.0 ± 9.4	31.1 ± 0.2
apigenin	3.1 ± 0.3	2.3 ± 0.2	2.6 ± 0.1	2.5 ± 0.1	3.8 ± 0.6	3.1 ± 0.4	3.3 ± 0.3	4.3 ± 1.2
resveratrol	56.8 ± 10.5	48.9 ± 2.9	40.1 ± 0.7	n.d.	56.1 ± 6.9	61.2 ± 10.9	41.3 ± 0.2	n.d.
cinnamic acid	9.5 ± 3.3	12.4 ± 5.1	5.5 ± 1.4	2.1 ± 0.1	10.2 ± 2.8	15.6 ± 8.3	20.2 ± 9.6	2.0 ± 0.1
rosmaniric acid	46.4 ± 0.8	34.0 ± 1.6	38.7 ± 1.3	n.d.	49.1 ± 2.3	49.4 ± 0.5	44.5 ± 1.9	n.d.
gallocatechin	180.5 ± 3.7	188.0 ± 1.3	43.3 ± 1.6	n.d.	178.9 ± 9.5	161.4 ± 4.6	141.6 ± 16.0	31.7 ± 2.8
taxifolin	344.4 ± 66.8	174.7 ± 73.6	106.1 ± 21.9	23.4 ± 1.0	353.1 ± 16.6	284.3 ± 10.8	117.3 ± 26.7	53.1 ± 17.9
quercetin	n.q.	n.q.	n.q.	n.q.	n.q.	n.q.	n.q.	n.q.
3,4 hydroxybenzoic acid	93.0 ± 7.7	76.7 ± 1.1	47.0 ± 3.4	18.4 ± 1.0	85.1 ± 4.4	75.1 ± 2.6	47.1 ± 7.4	12.5 ± 0.9
**Total**	**1627**	**1245**	**855**	**414**	**1612**	**1323**	**1102**	**535**

Values of phenolic compounds are expressed as concentration mean ± SD (mg/L) of three experiments. n.d., not detected; n.q., not quantified; LPBE, lyophilized pine bark extrac; MD, maltodextrin.

## Data Availability

Data sharing not applicable.

## Supplementary Materials

The following are available online at https://www.mdpi.com/2304-8158/10/2/328/s1, Table S1: Limit of detection (LOD) and limit of quantification (LOQ) for the different tested phenolic compounds. Table S2: Analysis of variance of the first-order polynomial models for spray drying encapsulates from pine bark by-product extracts.
