# Supplementary material for: Encapsulated Pine Bark Polyphenolic Extract during Gastrointestinal Digestion: Bioaccessibility, Bioactivity and Oxidative Stress Prevention
Source: Foods. 2021 Feb 4;10(2):328. doi: 10.3390/foods10020328 (PMC7913864; doi:10.3390/foods10020328)
Supplement: Supplementary file 1 [file foods-10-00328-s001.pdf]

Table S1. Limit of detection (LOD) and limit of quantification (LOQ) for the different tested phenolic compounds.

Compounds	LOD (mg/L)	LOQ (mg/L)	R^2
catechin	11.82	35.82	0.9989
vanilic acid	9.57	29.00	0.9996
gallic acid	23.31	70.63	0.9959
epicatechin + *p*-coumaric acid	12.78	38.71	0.9986
o-coumaric acid	11.75	35.60	0.9988
chlorogenic acid	17.71	53.67	0.9988
ferulic acid	31.19	94.53	0.9916
ellagic acid	30.65	92.88	0.9912
narginin	9.89	29.96	0.9992
hisperidin	36.87	111.74	0.9996
apigenin	5.31	70.63	0.9989
resveratrol	32.95	99.85	0.9909
cinnamic acid	13.99	42.40	0.9998
rosmaniric acid	14.42	43.71	0.9970
gallocatechin	13.38	38.98	0.9989
taxifolin	47.98	145.39	0.9917
quercetin	21.34	64.67	0.9961
3,4 hydroxybenzoic acid	9.60	29.07	0.9993

Table S2. Analysis of variance of the first-order polynomial models for spray drying encapsulates from pine bark by-product extracts.

Coefficient	MC	EE		
		AA_{ABTS}	AA_{FRAP}	TPC
Model	3.12**	92.78***	89.73***	62.38**
T	-0.21*	2.76*	-19.53***	2.74*
r	0.38**	5.00***	-3.54*	3.98*
F	0.11	6.30***	11.17***	4.15*
F * *T*	-	-7.75*	-0.22*	-
F * *r*	0.43**	-3.76**	-6.16**	-
T * *r*	-0.29*	6.37**	10.26***	-5.96**
T^2	0.80***	-	-	-15.96**
r^2	-	-	-	-10.3***
F^2	-	-	-	6.25*
R^2	0.906	0.934	0.933	0.856
R^2-*adj*	0.811	0.901	0.910	0.812
Eq.	$MC = 52.7 - 0.63\ T + 0.47\ r + 0.39\ F + 0.03\ F\ r - 2.89\ T\ r + 1.20\ T^2$	$AA_{ABTS} = 276.53 + 1.97\ T + 13.19\ r + 25.92\ F - 0.13\ F\ T - 0.25\ F\ r + 0.06\ T\ r$	$AA_{FRAP} = 54.33 - 0.28\ T - 12.43\ r + 9.45\ F - 3.71\ F\ T - 0.41\ F\ r - 0.10\ T\ r$	$TPC = 985.28 + 12.63\ T + 1.54\ r + 6.98\ F - 0.09\ F\ r - 0.04\ T^2 - 0.42\ r^2 + 0.63\ F^2$

Adjusted determination coefficient (R^2-*adj*) and determination coefficient (R^2) for evaluating model goodness-of-fit and model equations are also reported. *T*, temperature (°C); *r*, ratio lyophilized pine bark extract:Maltodextrin (-); *F*, flow rate (mL/min); *M*, moisture content (%); *E*, encapsulation efficiency (%); *TPC*, total phenolic content (%); *AA*, antioxidant activity measured by *ABTS* assay (%), and *FRAP* assay (%). * $p < 0.05$, ** $p < 0.01$, *** $p < 0.001$.
